# The Effect of Different Abutment Designs and Materials on Stress Distribution in Implants and Peripheral Bones: A Three-Dimensional Finite Element Analysis

**DOI:** 10.7759/cureus.64871

**Published:** 2024-07-18

**Authors:** Sreedevi Kondareddy, Sudheer N, Anulekha Avinash CK, Mahesh P, Dilip Jayyarapu, Akhila Devi Kakarlapudi

**Affiliations:** 1 Prosthodontics, Kamineni Institute of Dental Sciences, Nalgonda, IND

**Keywords:** frc, peek, zirconia, titanium, customized abutment, stock abutment, fea

## Abstract

Aims: To evaluate and compare the distribution of stress patterns around a single implant-supported crown with various abutment designs using different abutment materials.

Setting and design: This is an in vitro study to evaluate and compare stress distribution patterns.

Methods: The three-dimensional (3D) finite element models included four stock and four customized abutments made of Titanium, Zirconia, Fibre-Reinforced Composite, and Poly Ether-Ether Ketone (PEEK) attached to an end-osseous root form implant. The models were subjected to a 300 N vertical load at the central fossa and a 150 N oblique load at the centre of the lingual inclines of the buccal cusps of the mandibular molar crown designed on the model. Statistical analysis used: The stress distribution within the implant and the surrounding supporting structures was evaluated using finite element analysis.

Results: In all the models, stresses on the implants were observed to be concentrated in the neck of the implants in the first few threads. Irrespective of the abutment design, PEEK abutment transferred greater stresses to implants and Zirconia abutment transferred lesser stresses to implants. In the implants, the customized abutment showed lesser stress values than the stock abutment during oblique loading.

Conclusions: Stresses on implants and cortical bone can be reduced by using Zirconia as an abutment material compared to Titanium, Fibre-Reinforced Composite, and Poly Ether-Ether Ketone material. Customized abutments improve the load transfer between the prosthesis and the implant and the surrounding bone, lessen the micro-movement of the abutments, and distribute the stress more evenly across the implant's component parts.

## Introduction

In clinical dentistry, implant-supported prostheses have gained popularity as a therapy option due to functional, biological, and mechanical benefits as well as their long-term clinical success rates [1,2]. Implants lacking periodontal ligament are in direct contact with bone, and there is a decrease in proprioception, which may cause excessive stress on restorations and occlusal loads are immediately transmitted to the nearby bone structure [3,4]. The stress distribution in implants and surrounding bone is impacted by this interaction, which is one of the key elements influencing implant success [5]. A few examples of these parameters are the loading direction [6], material properties and design of the restorative crown or implant [7], and the energy or stress transmission between the implant and peripheral bone.

Enhancing the restoration techniques for implant therapy through research on the biomechanics of dental implants has grown in popularity. In response to the aforementioned issues, a number of researchers have worked to develop newer abutment designs [8], to maintain proper oral hygiene [9], that optimize the transfer of load to implant and surrounding bone from prosthesis, reduce micro-movement of the abutment, and reduce stress concentration in the implant components [10].

Currently, there are different designs of abutments available, however the impact of abutment design on the implant and surrounding bone of a single crown supported by an implant in the posterior region is minimal. This study set out to determine the maximum von Mises stress, the pattern of stress distribution, and the amount of stress buildup of an implant supported by a single crown with different abutment designs in the posterior area of the mandible.

## Materials and methods

**Figure 1:**
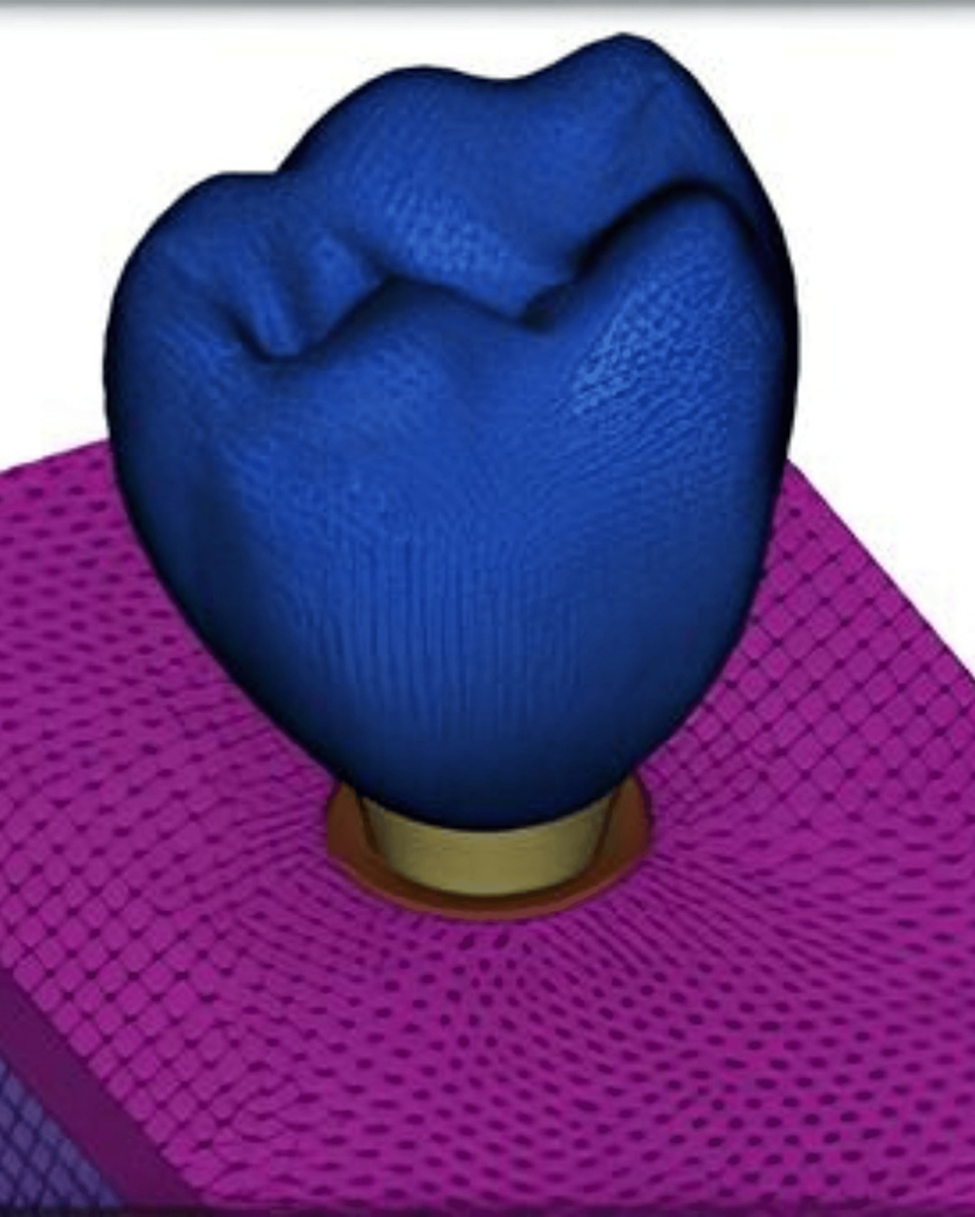
Meshed model of the mandibular molar

In the current study, model designing was done using the 3D CATIA program (Version 5 R20) (Dassault Systems, Paris, France) to construct two 3D geometric models, which were subsequently transformed into 3D finite element models using the Hyper mesh SOFTWARE 17.0 (Altair, Michigan, USA) tool. Analysis was done using ANSYS Software, version 16.2 (ANSYS, Inc., PA, USA). The study has been approved by the Institutional Ethical Committee (KIDS/IEC/2017/12). A finite element model of the mandibular section of bone was designed (Figure 1).

**Figure 2:**
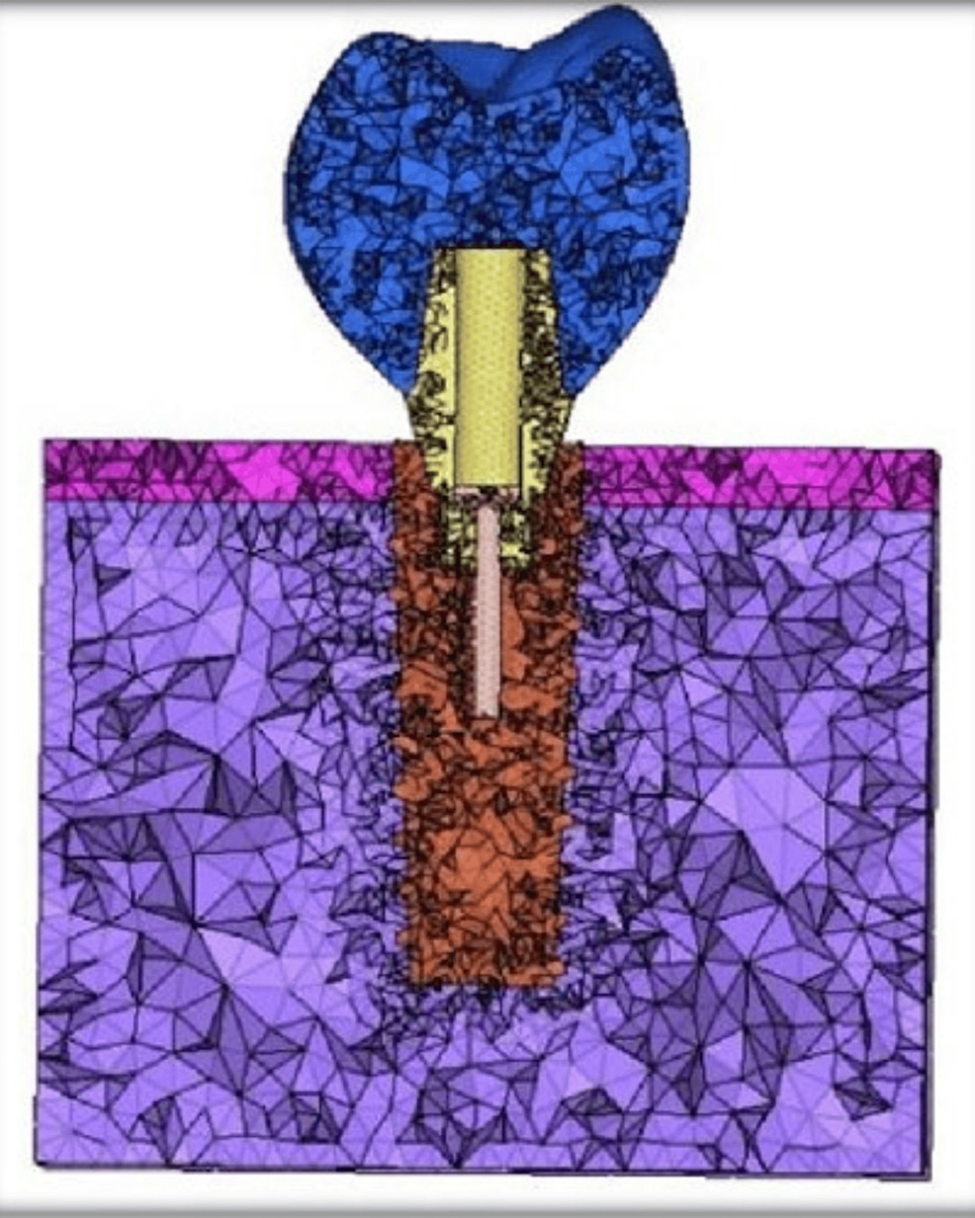
Meshed model of stock abutment along with abutment screw

**Figure 3:**
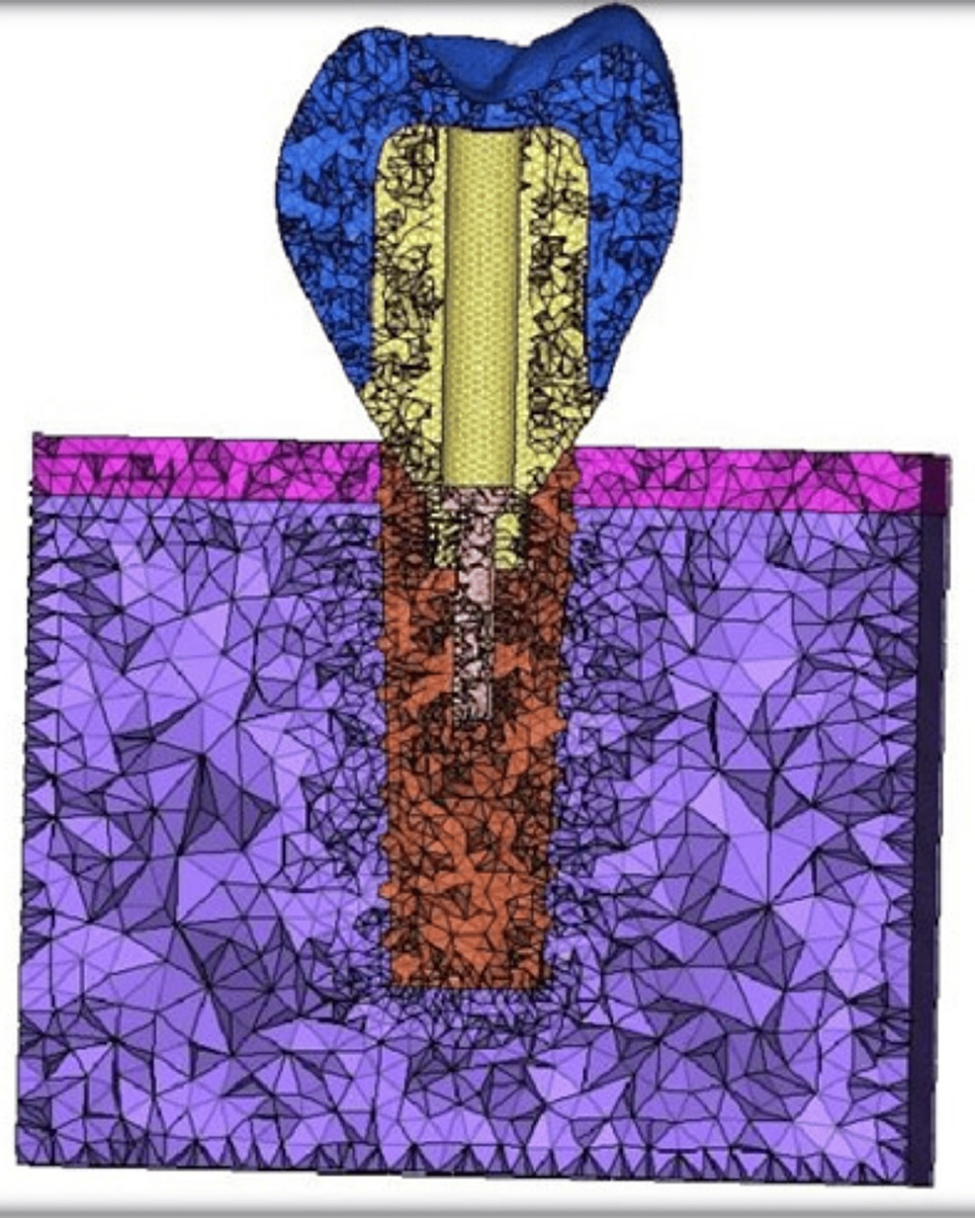
Meshed model of customized abutment along with abutment screw

The eight models included four stock abutments and four customized abutments made of Titanium (Ti), Zirconia (Zr), Fiber Reinforced Composite (FRC), Poly Ether-Ether Ketone (PEEK), attached to an end-osseous root from titanium implant replacing a mandibular molar restored with a zirconia crown. Two abutment models were employed in the analysis, one of which had a stock design (Figure 2) and another which had a customized design (Figure 3).

The implant fixture, abutment, abutment screw, crown, and bone (cortical and cancellous) were the five components of each 3D model. A bone block with a height of 12.5 mm, width of 10 mm, and cortical bone thickness of 2 mm surrounding the cancellous bone was modeled. Three-dimensional (3D) representations of an implant system at the bone level were designed utilizing the original implant component's standard tessellation language (STL) data. As an example of an implant fixture and abutment design that can be purchased commercially, the TS III implant series from OSSTEM Implant (Osstem Implant Co., Ltd., Seoul, Korea) was chosen. The specially made abutments were created. The FEM model was then created using the computer program Hyper mesh 17.0. All the models were constructed using three-dimensional 4-node tetrahedral elements. The number of nodes and elements for the models is mentioned in Table 1.

**Table 1:** Model description The number of nodes and elements for the models are given here.

Model description	Elements	Nodes
Implants with stock abutment	277335	53604
Implants with customized abutment	304248	58188

Anisotropic material properties of cortical and trabecular bone were obtained from the literature and adopted in the FE models. Furthermore, it was assumed that the Titanium, Zirconia, Fiber-reinforced composite (FRC), Poly Ether-Ether ketone (PEEK), and Zirconia components of the prosthetic crown and the implant were linearly elastic and isotropic (Table 2).

**Table 2:** The material properties used in the finite element analysis (FEA) model PEEK: Poly Ether-Ether Ketone.

Components	Elastic Modulus (G Pa)	Poisson’s ratio (ʋ)
Cortical bone	13.7	0.30
Cancellous bone	1.37	0.30
Titanium	110	0.35
Zirconia	210	0.30
Fiber reinforced composite	72.3	0.2
PEEK	3.5	0.36
Full contoured zirconia crown	210	0.30
Resin modified glass ionomer cement	6.3	0.3

**Figure 4:**
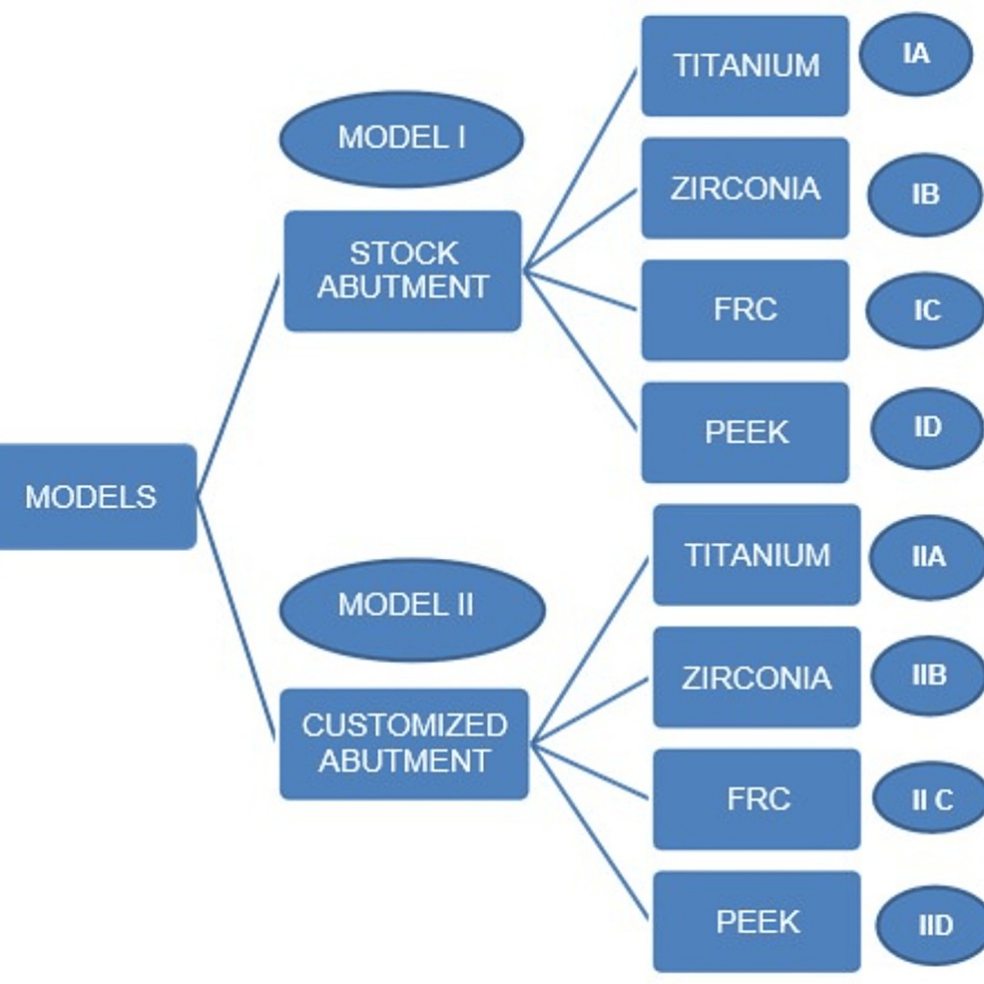
Flow chart of the models designed in the study

Two models designed with stock and customized abutments were incorporated with all the material properties. The flow chart of the models in the study is shown in Figure 4.

The models were subjected to static loads of 300N vertical load at the central fossa and 150 N oblique load at the center of the lingual inclines of buccal cusps of mandibular molar. Loading stresses were induced in the implant, abutment as well as on the surrounding cortical and cancellous bone. After analyzing the stress color plots, the maximum von Mises stress that was created in each supporting layer was compared and recorded.

## Results

In all the models described in the flow chart, the von Mises stresses (MPa) in implant, abutment, abutment screw, cortical bone in stock and customized abutment models under axial and oblique loading were tabulated (Table 3).

**Table 3:** A comparison of von Mises stresses (MPa) in implant, abutment, abutment screw, and cortical bone The table compares stock and customized abutment models under axial and oblique loading. PEEK: Poly Ether-Ether Ketone; FRC: Fibre-Reinforced Composite.

S.No	Component	Stock abutment		Customized abutment	
1	IMPLANT	Axial load (300 N)	Oblique load (150 N)	Axial load (300 N)	Oblique load (150 N)
	Titanium	71.55	265.25	73.75	182.917
	Zirconia	71.42	219.07	73.60	149.63
	FRC	83.04	312.419	73.83	209.74
	PEEK	132.39	522.502	113.78	309.852
2	ABUTMENT	Axial load (300 N)	Oblique load (150 N)	Axial load (300 N)	Oblique load (150 N)
	Titanium	111.84	397.72	203.48	186.806
	Zirconia	116.25	417.83	295.64	195.43
	FRC	100.83	369.86	191.67	160.709
	PEEK	100.121	369.07	65.93	157.71
3	SCREW	Axial load (300 N)	Oblique load (150 N)	Axial load (300 N)	Oblique load (150 N)
	Titanium	64.33	144.22	42.104	67.28
	Zirconia	52.68	120.67	35.59	64.87
	FRC	70.15	156.59	44.59	71.77
	PEEK	78.57	160.26	51.4	72.42
4	CORTICAL BONE	Axial load (300 N)	Oblique load (150 N)	Axial load (300 N)	Oblique load (150 N)
	Titanium	21.06	35.76	30.49	53.21
	Zirconia	15.45	33.09	23.95	52.58
	FRC	23.82	36.98	32.37	55.72
	PEEK	24.78	38.50	32.5	58.68

**Figure 5:**
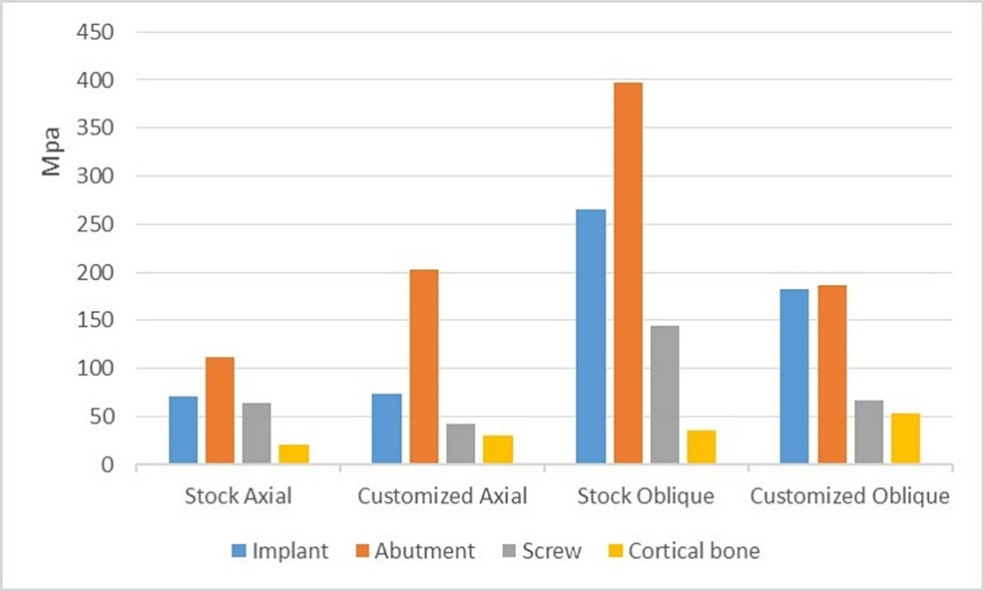
A graph showing von Mises stresses in titanium stock (IA) and customized abutment (IIA) models Von Mises stresses induced in the implant, abutment, abutment screw and cortical bone in titanium stock (IA) and customized abutment (IIA) models under axial and oblique loadings.

**Figure 6:**
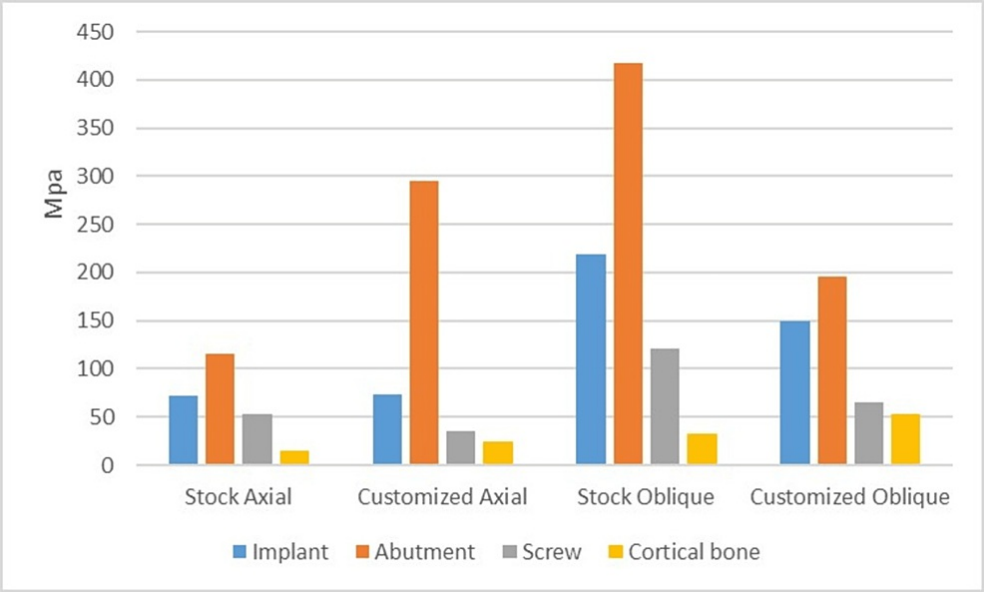
A graph showing von Mises stresses in Zirconia stock (IB) and customized abutment (IIB) models Von Mises stresses induced in the implant, abutment, abutment screw and cortical bone in Zirconia stock (IB) and customized abutment (IIB) models under axial and oblique loadings.

The data tabulated was plotted against the graphs for all the models, graphs showing the stresses on the titanium abutment models (Figure 5) and a graph showing the stresses on the Zirconium abutment models (Figure 6). 

**Figure 7:**
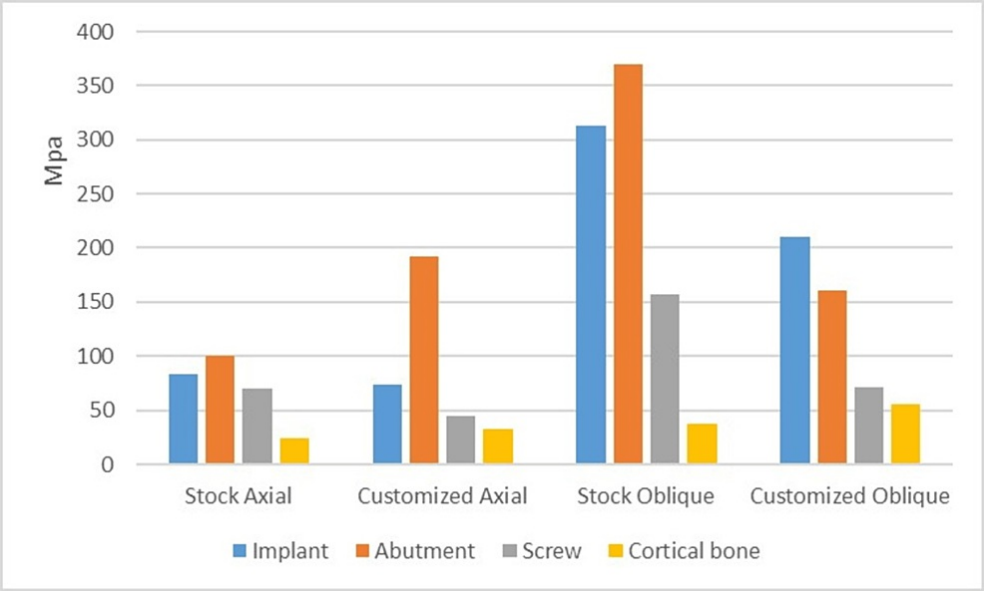
A graph showing von Mises stresses in FRC stock (IC) and customized abutment (IIC) models Von Mises stresses induced in the implant, abutment, abutment screw and cortical bone in Fibre-Reinforced Composite (FRC) stock (IC) and customized abutment (IIC) models under axial and oblique loadings.

A graph showing the stresses on the FRC abutment models (Figure 7). 

**Figure 8:**
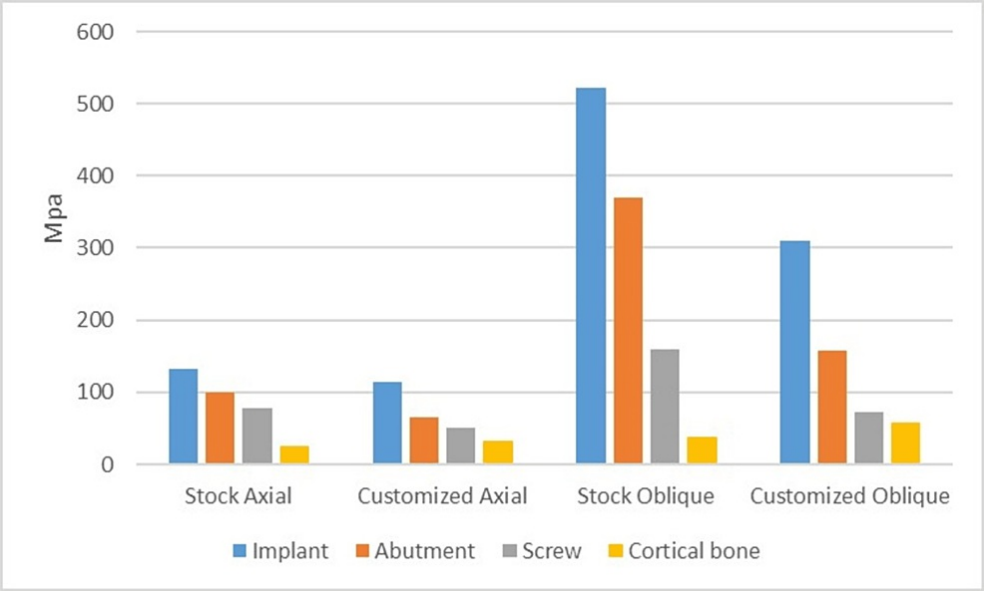
A graph showing von Mises stresses in PEEK stock (ID) and customized abutment (IIC) models Von Mises stresses induced in the implant, abutment, abutment screw and cortical bone in Poly Ether-Ether Ketone (PEEK) stock (ID) and customized abutment (IID) model under axial and oblique loadings.

A graph showing the stresses on the PEEK abutment models (Figure 8).

**Figure 9:**
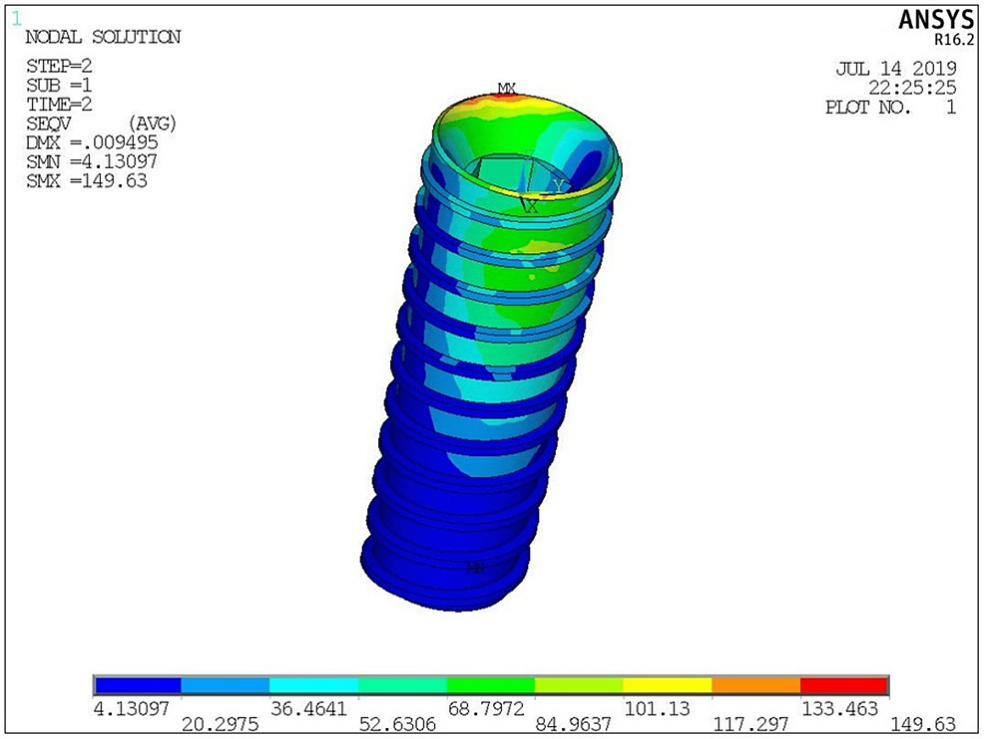
Von Mises stresses induced in the implant

**Figure 10:**
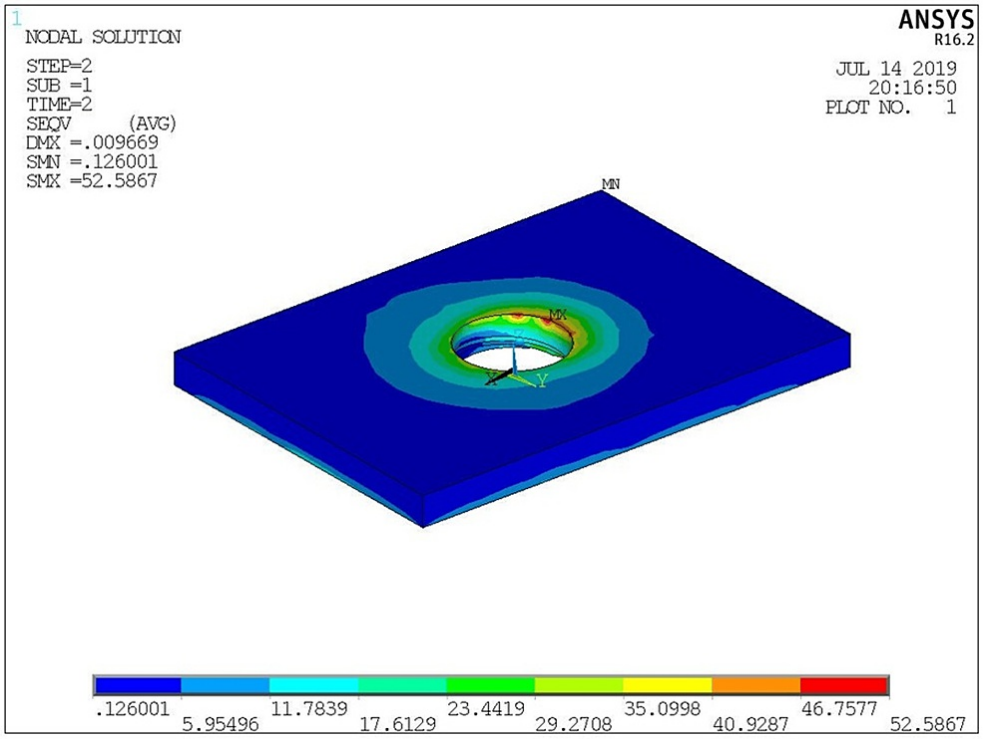
Vmax stresses on cortical bone

Vmax stress color plot patterns with load application in models at implant, cortical bone are shown in (Figure 9) and (Figure 10) respectively.

The ANSYS software was used to analyze the stresses on each model. On the model, the stresses were represented by various colors. Stresses were evaluated by looking at the hues. There was a specific range of stress levels for each hue. The maximum and minimum levels of stress contained all the colors from blue to red. In all the models, stresses on the implants were observed to be concentrated in the neck of the implants in the first few threads. It was observed that the maximum stress generated at the implant level was in the PEEK material under both axial and oblique loads. Customized abutment design showed lesser stress values than stock abutment during oblique loading in the implants.

## Discussion

Implant dentistry has established itself as the epitome of modern dentistry. Titanium implants are now used in dentistry with high success rates. In our study, titanium implants were used in all groups. Biomechanics has a significant role in the clinical success of dental implants. A homogeneous distribution of stress to the bone is required for maintaining the implant‑bone combination for a long time [11].

In order to meet the requirements for function, appearance, and biology, implant abutments should be constructed from biocompatible materials with sufficient mechanical qualities. To avoid issues such as screw loosening, bone loss, and abutment fractures during function, abutments should fit the implants precisely and passively. The right emergence profile is also necessary for implant abutments to support the surrounding soft tissue and achieve the best mucogingival esthetics. Custom or stock abutments are available for implants. Implant abutments can be either stock abutments or customized abutments. Kim et al. suggested that the model with the customized abutment can offer improved biomechanics for a single crown restoration supported by an implant [12]. In order to maintain good oral hygiene optimize the load transfer from prosthesis to implant and surrounding bone [8], lessen abutment micro-movement, and lower stress concentration in the implant components, a number of researchers have contributed to the development of new abutment designs. Abutment materials available, however, are varied. Among the materials available, Titanium, Zirconia, FRC, and PEEK are used widely each with their own advantages and disadvantages.

Zirconia is a biocompatible material that has optimal aesthetics and well-established characteristics, including decreased bacterial growth, strong osseointegration, and a bone-to-implant contact surface resembling titanium and hence zirconia could potentially represent a good substitute for titanium as an implant and abutment material [13]. FRC abutment is easy to prepare for a pillar of a crown without considerable heat generation. Synthetic thermoplastic polymer PEEK has been utilized as a biomaterial for medical purposes since the 1980s. It has a high mechanical performance. PEEK biomaterials have garnered interest lately as an alternative to metal alloys for usage as coating, computer-aided design-computer-aided manufacturing (CAD-CAM)-milled framework, implant, and abutment materials [14]. Presently, implants made of pure PEEK and carbon fiber-reinforced PEEK (CFR-PEEK) are accessible [15]. Furthermore, the stress effects on peripheral bone may be lessened due to the compatibility of the PEEK biomaterial's elastic modulus with bone [16]. The biomechanical interactions between the implants and the supported structures supply us with the knowledge of the load transmission process.

An analytical approach for simulating structures and analyzing force-displacement and internal stress relationships is the finite element method. According to this technique, a structure is made up of a number of geometric structural pieces that are joined at nodes. The mechanical properties of the real material each constituent represents are applied to it. Stress contours and values are calculated using equations for nodal displacements resulting from applied loads. In the present study, the model properties were considered homogeneous, isotropic, and linearly elastic which means that the material properties are identical in all directions, and only two independent variables (elasticity modulus and Poisson’s ratio) were defined. The modelling in the current study was done using 3D software called 3D CATIA programme, after which it was exported to an analysis package. The finite element software, ANSYS Workbench was used to analyze the model. The models were processed in ANSYS to generate the meshed structure. Meshing divided the entire model into smaller elements and the boundaries were defined. The loads to be applied were defined, and then the stress analysis was completed by the incorporation of material properties which were determined from values obtained from the literature.

The present study was performed and undertaken by the Department of Prosthodontics at Kamineni Institute of Dental Sciences, Sreepuram, Narketpally, with assistance in design and FEA from Concurrent Analysis Pvt. Ltd, Hyderabad. The present study was conducted to evaluate the stresses in the implant, abutment, abutment screw and cortical bone with Titanium, Zirconia, FRC and PEEK stock and customized abutments under simultaneous and progressive loading in order to understand stress patterns generated along the various components of the 3D model. Prosthesis materials and designs are the factors that greatly influence the stress distribution in the bone around implants [17].

Implant

In all the models, stresses on the implants were observed to be concentrated in the neck of the implants in the first few threads. Similar results were observed in a study conducted by Poovarodom et al., to examine the distribution of stress in dental implant supported restorations in a mandibular first molar using different implant abutment designs [18].

When all models were examined in accordance with the results obtained in this study, it was observed that the use of PEEK abutment, irrespective of the abutment design transferred greater stresses to implants. According to Rodrigo et al., CFR-PEEK showed an increased concentration of stress in the implant neck as a result of increased deformation and lower stiffness compared to titanium [19]. Zirconia abutment, irrespective of abutment design, transferred lesser stresses to implants than titanium, FRC, and PEEK. These results are similar to a study conducted by Cagler et al., who examined the von Mises tensile and compressive stresses that occur on implants, surrounding bone, and abutments in three anterior maxilla simulations using three-dimensional (3D) finite element analysis (FEA) [20].

According to Tekin et al., the use of titanium abutments with more elastic modulus reduced the stress on the implant and also it was observed that the use of different prosthetic materials can change the stresses in the implant system [11]. The oblique forces transferred greater stresses than axial forces to implants irrespective of the abutment design. According to Carlos et al., oblique loads lead to higher stress concentration in the cortical peri-implant bone, in the implant and in the abutment screw [5]. Customized abutment design showed lesser stress values than stock abutment during oblique loading in the implants. According to Poovarodam et al., better biomechanics is provided by customized abutment and there was a continuous stress distribution pattern with a lower maximum stress accumulation in the implant fixture [18]. The greater detrimental forces for the implant osseointegration and maintenance are the lateral forces acting on it which, when over a threshold can lead to angular bone loss and peri-implantitis, and implant failure.

Cortical bone

In the presence of cortical bone, most of the loads transferred by the implant to the bones were met by the cortical bone itself, leading to a much lower amount of stress being transmitted to the trabecular bone. The highest stress values in the bone were detected in areas where the implant first contacted the cortical bone in the neck region.

When compared to zirconia abutments, a larger value of stress concentration on cortical and spongy bone is exhibited by the titanium abutment. The overall stress and distortion of the implant decrease with increasing abutment material rigidity. This suggested that when the stiffness of the abutment material increased, the implant material absorbed less energy. According to Anwar et al, when compared with alumina and zirconium types, titanium abutment shows a high value of stress concentration on cortical and spongy bone [21]. Shinya et al. examined the stress and strain that were caused on the implant and the surrounding bone tissue by two distinct implant materials: titanium (Ti) and experimental fiber-reinforced composite (FRC), they concluded that higher strain was detected in the cortical bone around the FRC implant than around the Ti implant [22]. 

According to Rodrigo et al., CFR-PEEK showed an increased concentration of stress in the implant neck as a result of increased deformation and lower stiffness compared to titanium [19]. Papavasiliou et al. showed that oblique loads could increase stresses by up to 10-fold. Similarly, the results of the present study indicated that oblique loading forces caused the highest maximum stress values [23]. The implant fixture's first thread showed the highest maximum stress. During mastication, a broad range of loads between 17 and 450 N are seen [24]. For long-term survival, occlusal interferences must be removed and the ideal occlusal connection must be established.

The abutment material's reduced stiffness means that it will absorb less energy and shift more load onto the model's subsequent sections. They are in agreement with those of Caglar et al.'s study. Stress values can change significantly when the abutment material is changed [20]. Compared to softer abutment materials, stiffer abutment materials absorb more energy. The least stresses were observed in the customized abutment models. This is because, in comparison to the prefabricated abutment, the customized abutment has a larger collar. A larger collar can more effectively disperse stress. According to Mammadzada et al., it was determined that there was a direct correlation between the abutment's shape and the distribution of stress on the abutment, implant fixture, and bone [25]. The abutment screw fracture is a frequent problem in the clinical field. The increased stress concentration shown in the oblique load application can make the abutment screw more susceptible to loosening or fracture.

In the present study several assumptions and simplifications have been made with regards to the model generation and material properties. But the models may deviate from reality in several aspects. This study has mainly focused on the abutment designs and the material aspects of the models rather than other factors that may affect the stress distribution. Therefore, the inherent limitations of this FEA must be acknowledged.

Limitations of the study

The biomechanics of stress transfer in dentistry has been widely studied through the use of FEM studies. The supporting tissues were taken to be homogeneous, consistent, and linearly elastic structures; the perfect osseointegration of the implant with bone was also assumed; and the inherent complexity of the host made it extremely difficult to fabricate an appropriate model. The study's results may not accurately reflect actual values due to these constraints, but they may nonetheless highlight the benefits and variations in stress between different abutment designs. Hence, quantitative data of the study may not be applied directly to clinical practice. So, a long-term in vivo study to support the above tests must be carried out.

## Conclusions

Considering the results obtained, it can be proposed that the use of customized abutment designs provides better biomechanics than the stock abutment designs. Among the abutment materials used in the present study, Zirconia as an abutment material can reduce the stresses on the implants, abutment screw and cortical bone when compared to Titanium, FRC, and PEEK abutment material in that order as it is a biocompatible material that has optimal aesthetic properties, well-established characteristics including decreased bacterial growth, a bone-to-implant contact surface like titanium and fine osseointegration. These customized abutments help to minimize abutment micro-movement, decrease stress concentration in the implant components, optimize load transmission from prosthesis to implant and surrounding bone and maintain good oral hygiene. Masticatory stresses are transferred from the restorative crown to the abutment and the abutment screw before being transmitted to the implant and surrounding bone. Therefore, the ability of an abutment to both absorb and transmit these stresses is important.
